# The feasibility of epidemiological research on prostate cancer in African men in Ibadan, Nigeria

**DOI:** 10.1186/s12889-015-1754-x

**Published:** 2015-04-26

**Authors:** Ilir Agalliu, Akin O Adebiyi, David W Lounsbury, Oluwafemi Popoola, Kola Jinadu, Olukemi Amodu, Suvam Paul, Adebola Adedimeji, Chioma Asuzu, Michael Asuzu, Olufemi J Ogunbiyi, Thomas Rohan, Olayiwola B Shittu

**Affiliations:** Department of Epidemiology and Population Health, Albert Einstein College of Medicine, 1300 Morris Park Avenue, Belfer Building, Room 1315-B, Bronx, NY 10461 USA; Department of Urology, Albert Einstein College of Medicine, Bronx, NY USA; Department of Community Medicine, Clinical Epidemiology Unit, College of Medicine, University of Ibadan, Ibadan, Nigeria; Institute of Child Health, College of Medicine, University of Ibadan, Ibadan, Nigeria; Department of Radiotherapy, Psycho-Oncology Unit, College of Medicine, University of Ibadan, Ibadan, Nigeria; Department of Pathology, College of Medicine, University of Ibadan, Ibadan, Nigeria; Department of Surgery, Urology Unit, College of Medicine, University of Ibadan, Ibadan, Nigeria

**Keywords:** Prostate cancer, Epidemiologic study, Case–control study, Feasibility study, Nigeria, African men

## Abstract

**Background:**

Men of African descent have the highest incidence and mortality rates of prostate cancer (PrCa) worldwide. Notably, PrCa is increasing in Africa with Nigerian men being mostly affected. Thus, it is important to understand risk factors for PrCa in Nigeria and build capacity for cancer research. The goals of this study were to determine the feasibility of conducting an epidemiological study of PrCa and to obtain preliminary data on risk factors for PrCa in Nigeria.

**Methods:**

A case–control study (50 cases/50 controls) was conducted at the University College Hospital (UCH) in Ibadan, Nigeria, between October 2011 and December 2012. Men aged 40 to 80 years were approached for the study and asked to provide informed consent and complete the research protocol. Logistic regression models were used to examine associations between demographic, social and lifestyle characteristics and risk of PrCa.

**Results:**

The participation rate among cases and controls was 98% and 93%, respectively. All participants completed a questionnaire and 99% (50 cases/49 controls) provided blood samples. Cases had a median serum diagnostic PSA of 73 ng/ml, and 38% had a Gleason score 8–10 tumor. Family history of PrCa was associated with a 4.9-fold increased risk of PrCa (95% CI 1.0 - 24.8). There were statistically significant inverse associations between PrCa and height, weight and waist circumference, but there was no association with body mass index (kg/m^2^). There were no associations between other socio-demographic and lifestyle characteristics and PrCa risk.

**Conclusion:**

This feasibility study demonstrated the ability to ascertain and recruit participants at UCH and collect epidemiological, clinical and biospecimen data. Our results highlighted the advanced clinical characteristics of PrCa in Nigerian men, and that family history of PrCa and some anthropometric factors were associated with PrCa risk in this population. However, larger studies are needed to better understand the epidemiological risk factors of PrCa in Nigeria.

## Background

Prostate cancer (PrCa) is the most commonly diagnosed solid tumor in men and the second leading cause of cancer-related deaths in developed countries [1-3]. This cancer has a complex, multi-factorial etiology and both genetic and environmental/lifestyle factors contribute to its development [4]. Although extensive research has been conducted to uncover risk factors for PrCa, the only well-established epidemiological risk factors are age, race/ethnicity and family history of PrCa [2,5,6]. There is a large variation in PrCa rates internationally with men of African and Afro-Caribbean descent having the highest incidence of and mortality from this cancer [7-9]. Family history of PrCa among first-degree relatives is associated with a significant, 2- to 3-fold increased risk of PrCa [10,11], and both early ages at diagnosis and multiple affected family members are strong predictors of risk in relatives.

The role of other putative factors in PrCa etiology including smoking, alcohol, obesity, diet, and androgens is inconclusive with some studies reporting positive associations, and others no associations [12-20]. Epidemiologic studies indicate that higher intake of foods rich in antioxidants such as vitamins C and E, lycopene, β-carotene, selenium and cruciferous vegetables are inversely associated with PrCa risk, whereas consumption of foods that may increase oxidative stress, including saturated fats and red meat is associated with increased risk [13,16,21-28]. Lastly, body mass index (BMI)/obesity have been positively associated with PrCa, especially with risk of advanced grade/stage cancer [20,29,30]. Although the large international variation in PrCa incidence and mortality rates could partly be due to differences in age distribution, access to health care, and screening services, cultural and other factors including genetic susceptibility, and rapid changes in diet and lifestyle factors, particularly in developing countries, may also contribute to this disparity [31-33].

Data from the GLOBOCAN program of the *International Agency for Research on Cancer* (IARC) show that PrCa is also the leading cancer in sub-Saharan African men, accounting for 14% of all cancer diagnoses and 12% of all cancer-related deaths [34,35]. Men in Nigeria suffer the highest burden from PrCa with estimated annual age-adjusted incidence and mortality rates of 22.7 and 18.6 per 100,000, respectively. This accounts for 18.2% and 17.7% of all cancer-related diagnoses and deaths, respectively, in men in this region [7,34,35]. Given that Nigeria is the most populous country in Africa with an estimated population of 160 million in 2012, the rates and percentages above translate to an enormous burden in absolute numbers of men affected by PrCa.

The few published case-series reports on PrCa in Nigeria demonstrate an increasing trend in incidence over a 30-year period raising it from the 8th to the 1st ranked cancer [36-38]. Furthermore, data from previous reports [37,39-43] show that 65% to 80% of all PrCa patients in Nigeria are diagnosed with advanced tumor stage and grade, and men affected by PrCa in this country have the lowest 5-year survival rates in the world. While the above data demonstrate the need to urgently investigate PrCa in Nigeria, the reported numbers are likely to be an underestimate of the true burden of PrCa, since most of the reports are based on limited population and incomplete cancer registry data [9].

Therefore, it is important to conduct large studies to better understand epidemiological risk factors for PrCa morbidity and mortality in Nigerian men as well as to build capacity for cancer research and biospecimen collection and storage in Nigeria. As an initial step toward this goal, a collaboration was initiated between Albert Einstein College of Medicine in New York City and the University College Hospital (UCH) in Ibadan, in order to assess the feasibility of conducting a small, hospital-based case–control study of PrCa at the latter institution. The primary goals of the study were to assess participation rates of PrCa patients and controls at UCH, to investigate the feasibility of collecting, storing and analyzing bio-specimens in partnership with UCH, and to obtain preliminary estimates on the prevalence of various potential risk factors for PrCa in Nigerian men. An additional objective was to start building capacity and infrastructure to conduct high quality epidemiological and clinical research studies on cancer in Nigeria.

## Methods

### Study population and data collection

A hospital-based case–control study of PrCa (50 cases/50 controls) was conducted at University College Hospital (UCH), a major tertiary hospital and academic medical center established in 1957 in Ibadan, Nigeria. The recruitment of participants started in October 2011 and ended in December 2012; however, the active recruitment period was 13 months because of several interruptions during this period due to strikes that affected hospital operations. Ethical approval for the study was sought and obtained from the Institutional Review Boards (IRBs) of the Albert Einstein College of Medicine in New York City and the University College Hospital in Ibadan, Nigeria, before recruitment started.

### Ascertainment of participants

*Cases* were incident prostate cancer patients, aged 40 to 80 years at diagnosis, who were admitted and/or undergoing treatment for PrCa at UCH during the ascertainment period. They were identified by reviewing medical records and patients’ appointments, primarily from Urology. All patients with a histologically-confirmed prostate tumor of any grade were eligible for the study; a case was considered incident if the initial diagnosis of PrCa was within 6 month-period before contact with the patient. Cases underwent a chart review to confirm their PrCa diagnosis and tumor characteristics. Men younger than 40 years of age were excluded because invasive PrCa is very rare in this group. We also excluded men aged over 80 years because of potential comorbidity issues and the challenge in conducting interviews in this age group.

The designated resident doctor at Urology and the study coordinator reviewed medical records on a weekly basis to identify potentially eligible patients and their appointment dates. Patients were approached during their Urology clinic visit or within 72 hours of admission to the hospital by the study coordinator or the senior registrar in Clinical Epidemiology in order to explain the purpose of the study, and obtain informed consent. The informed consent assessed willingness to participate at different levels: Level 0- refusal to participate in any aspect of the study (administer short debriefing survey to inquire about reason(s) for non-participation); Level 1- willingness to complete a structured interview that collected information about PrCa risk factors as well as give permission for review of patient’s medical records; Level 2- Level 1 and provide a blood specimen; or Level 3- Level 1 and provide a buccal cells sample (for patients that refused to provide a blood sample). The informed consent and study instruments were provided in either English or Yoruba (the most prevalent indigenous language in Ibadan) to accommodate participants who did not speak English fluently, although English is the official language spoken in Nigeria.

A total of 52 potentially eligible cases were identified during the ascertainment period, but 51 were approached for the study (one patient died before he was contacted). Of the 51 eligible patients, 50 (98%) agreed to participate in the study. All 50 enrolled prostate cancer patients provided written informed consent and elected Level 2 participation (i.e., completed a structured interview, gave permission to have their medical records reviewed, and provided a blood sample for genomic DNA).

*Controls* were men aged 40 to 80 years, who did not have a prior history of or symptoms related to PrCa, and they were frequency-matched to cases by 5-year age group. The controls were recruited from hospital departments and services that were not linked to Urology, namely Orthopedics, Ophthalmology, Internal Medicine and the Hypertensive Clinic, during the same ascertainment period as that for the cases. The study coordinator and medical residents reviewed medical records on a weekly basis to identify potential control participants. Eligible controls were approached at clinic visits or within 72 hours of admission to the hospital by the study coordinator and designated senior registrar in Clinical Epidemiology to explain the purpose of the study, and obtain informed consent. The informed consent process used was similar to that described for cases in order to assess different levels of participation; the only difference was that controls were asked to provide an additional blood sample (8 ml vial in a red top tube) for separation of serum that was used to measure their serum PSA levels.

A total of 54 eligible controls were approached for the study during the recruitment period. Of these, 50 (93%) agreed to participate, provided written informed consent, and completed the structured interview. Of the 50 enrolled controls, 99% (49/50) provided blood samples for serum and genomic DNA (one patient refused to provide blood and mouthwash).

### Questionnaire data and specimen collection

All enrolled participants (50 cases/50 controls) completed a structured questionnaire with a male interviewer at the time of recruitment, after signing the informed consent form. The questionnaire collected information on demographic, anthropometric, lifestyle and social characteristics, information about family size and structure, family history of PrCa, as well as other cancers in first- and second-degree relatives, and medical history, including frequency of major comorbid conditions and physician visits over the past five years. We also assessed dietary patterns and habits in the year before interview. Information about PrCa was collected for the cases by inquiring about signs and symptoms preceding cancer diagnosis, the time interval between any symptoms and the diagnosis date, and how the cancer was first detected.

After the interview, both cases and controls provided a blood sample that was collected in one 6 ml green top tube (heparin) and that was used to extract genomic DNA for future studies. In controls, we collected an additional vial of blood in an 8 ml red top tube to separate serum, which was used to measure their serum PSA at the time of recruitment. We did not measure serum PSA levels in cases since blood samples were collected after PrCa diagnosis and treatment. However, for PrCa cases information on diagnostic serum PSA levels was extracted from medical records. All blood samples were collected by a trained phlebotomist and transported to the laboratory at UCH in cold containers within two hours of obtaining samples. DNA was extracted from white blood cells (the buffy coat layer) following the laboratory instructions included with the Nucleon BACC2 Kit from GE Health Care Europe. Total serum PSA was measured using VIDAS® tPSA assay in controls. All blood, serum and DNA samples were stored in a −20°C freezer in laboratories at UCH.

### Statistical analyses

We compared the demographic, social, lifestyle and dietary factors between cases and controls using Student t-tests (for continuous normally-distributed variables), Wilcoxon rank-sum test (for continuous non-normally-distributed variables) and chi-square tests (for categorical variables). All tests were two-sided using a significance level α = 0.05. Unconditional logistic regression models were used to estimate odds ratios (ORs) and 95% confidence intervals (CI) for the associations between various exposures of interest and PrCa risk, with adjustment for age [44]. Since the sample size for this feasibility study was small we did not have sufficient statistical power to conduct multivariate modeling or stratified analyses.

## Results

Table 1 provides a description of demographic and social characteristics of the 50 PrCa patients and 50 controls recruited into the study. Participants were, on average, 69 years old and the majority was of Yoruba ethnicity (88% of cases and 96% of controls). A first-degree family history of PrCa was associated with a 4.9-fold increased risk of PrCa (95%CI 1.0 - 24.8). There was only one control who reported a second-degree relative with PrCa, but none of the cases did. With regard to social factors, controls were more likely to be professionals with slightly higher college education in comparison to cases, although none of these characteristics was statistically significantly different between the two groups. The average annual income in this population was low (equivalent to US $20-$50) since the majority of participants were retired. However, it was similar between cases and controls.

**Table 1:** **Selected demographic and social characteristics of prostate cancer cases and controls**

**Characteristics**	**Cases (n = 50)**		**Controls (n = 50)**		**P**	OR*	**95% CI**	
	**n**	**%**	**n**	**%**				
Age at interview (years)					0.99			
45 - 59	5	10.0	5	10.0				
60 - 69	20	40.0	20	40.0				
70 - 80	25	50.0	25	50.0				
Ethnic background					0.14			
Yoruba	44	88.0	48	96.0		1.00	-	
Other†	6	12.0	2	4.0		3.42	0.63	18.62
First-degree relative with prostate cancer‡					0.046			
No	42	84.0	48	96.0		1.00	-	
Yes	8	16.0	2	4.0		4.87	0.96	24.76
Marital status					0.15			
Married	50	100.0	48	96.0				
Separated/Divorced	-		2	4.0				
Nr of biological childrenξ mean sd	7.16	3.38	6.26	3.03	0.16	1.10ξ	0.96	1.26
Religion/Faith					0.53			
Christian	30	60.0	33	66.0		1.00	-	
Muslim	19	38.0	17	34.0		1.26	0.55	2.91
Traditional	1	2.0	-					
Education					0.26			
None or <4 yrs	7	14.0	8	16.0		1.00	-	
4-18 yrs	27	54.0	19	38.0		1.63	0.50	5.31
College degree/Graduate	16	32.0	23	46.0		0.80	0.24	2.69
Employment status					0.14			
Currently employed	13	26.0	20	40.0		1.00		
Retired	37	74.0	30	60.0		2.03	0.82	5.03
Primary occupation					0.43			
Professional	11	22.0	15	30.0		1.00	-	
Managerial, technical or administrative	14	28.0	15	30.0		1.27	0.44	3.70
Operator, fabricator or laborer	16	32.0	8	16.0		2.73	0.86	8.65
Artisan	5	10.0	6	12.0		1.13	0.27	4.73
Farmer	4	8.0	6	12.0		0.91	0.21	4.01
Income (Nigerian Naira)					0.67			
<20,000	19	38.0	15	30.0		1.00	-	
20,000-50,000	18	36.0	19	38.0		0.75	0.29	1.90
>50,000	13	26.0	16	32.0		0.64	0.24	1.75

*OR and 95% CI were estimated from logistic regression models adjusted for age; OR = 1.00 refers to the reference category.

^†^Other ethnicities include Benin, Ebira, Edo, Hausa, Ibo, Ibibio, Ofemai.

^‡^First-degree relative includes father, brother(s) or son(s).

^ξ^OR and 95% CI are expressed per one unit increase (number of biological children).

We compared several anthropometric, lifestyle and dietary characteristics between PrCa patients and controls (Table 2). There were statistically significant inverse associations between risk of PrCa and height (OR = 0.91, 95% CI 0.86-0.97; per one cm increase), weight (OR = 0.97, 95% CI 0.94-1.00; per one kg increase; borderline significant), and waist size (OR = 0.91, 95% CI 0.87-0.96; per one cm increase). However, there was no difference in body mass index (BMI) between the two groups. The proportion of men who had lost 5 kg or more in the past year was higher in cases in comparison to controls (52% vs. 16%; p < 0.0001), which could potentially be attributed to cachexia due to cancer.

**Table 2:** **Anthropometric, lifestyle and dietary characteristics among prostate cancer cases and controls**

**Lifestyle characteristics**	**Cases (n = 50)**		**Controls (n = 50)**		**P**	OR*	**95% CI**	
Height (cm); mean sd	163.02	9.6	168.87	6.89	0.001	0.91^†^	0.86	0.97
Weight (kg); mean sd	67.84	12.10	72.35	11.85	0.06	0.97^†^	0.94	1.00
Waist size (cm); mean SD	80.44	9.33	88.92	10.53	<0.0001	0.91^†^	0.87	0.96
Body mass index (BMI; kg/m2) mean sd	25.68	0.68	25.33	0.51	0.69	1.02^†^	0.93	1.12
BMI category; n %								
<25	21	42.0	25	50.0	0.72	1.00	-	
25 - 29.9	21	42.0	18	36.0		1.39	0.59	3.28
≥30	8	16.0	7	14.0		1.35	0.42	4.36
Weight change ≥5 kg in last year					<0.0001			
No change	21	42.0	41	82.0		1.00	-	
Increased	3	6.0	1	2.0		6.08	0.59	62.59
Decreased	26	52.0	8	16.0		6.78	2.56	7.97
Moderate physical activity; n %					0.24			
Never or 1–3 times / month	13	26.0	19	38.0		1.00	-	
1- 6 times / week	13	26.0	15	30.0		1.26	0.45	3.57
Every day	24	48.0	16	32.0		2.19	0.85	5.67
Cigarette smoking status, n %					0.32			
Never smoker	29	58.0	33	66.0		1.00		
Former smoker Quit ≤ 27 years‡	11	22.0	7	14.0		1.79	0.61	5.23
Quit > 27 years‡	10	20.0	7	14.0		1.67	0.56	5.00
Current smoker	-	-	2	4.0		-		
Pack-years of smoking, n %					0.65			
Non-Smoker	29	58.0	33	66.0		1.00	-	
1-9.9	12	24.0	9	18.0		1.46	0.54	3.95
≥10	6	12.0	7	14.0		0.94	0.28	3.10
Alcohol consumption, n %					<0.0001			
Never user	7	14.0	19	38.0		1.00	-	
Former user	39	78.0	20	40.0		5.20	1.86	14.56
Current user	3	6.0	10	20.0		0.82	0.16	3.84
Missing	1	2.0	1	2.0				
Years since stopped drinking (former users); mean sd	16.57	2.17	26.68	2.91	0.008	0.94^†^	0.89	0.98
Drinks/week (current and former users); mean sd	1.27	0.92	3.91	5.41	0.014	0.72^†^	0.49	1.06
**Dietary intake**	**n**	**%**	**n**	**%**	**P**	OR*	**95% CI**	
Red meat, n %					0.72			
Never	8	16.0	7	14.0		1.00	-	
1- 3 times/month	7	14.0	11	22.0		0.56	0.14	2.30
1- 4 times/week	18	36.0	16	32.0		0.99	0.28	3.43
≥1 times/day	16	32.0	16	32.0		0.88	0.25	3.04
Pork, n %					0.40			
Never	48	96.0	46	92.0		1.00	-	
1- 3 times/month	2	4.0	4	8.0		0.48	0.08	2.75
Chicken, n %					0.15			
Never	16	32.0	7	14.0		1.00	-	
1 - 3 times/month	20	40.0	25	50.0		0.35	0.12	1.01
1 - 4 times/week	14	28.0	17	34.0		0.36	0.12	1.12
Smoked foods, n %					0.24			
Never	8	16.0	7	14.0		1.00	-	
1 - 3 times/month	9	18.0	18	36.0		0.44	0.12	1.59
1 - 4 times/week	27	54.0	20	40.0		1.18	0.37	3.80
≥1 times/day	6	12.0	5	10.0		1.05	0.22	5.02
Fish, n %					0.68			
1- 3 times/month	5	10.0	4	8.0		1.00	-	
1 - 4 times/week	27	54.0	24	48.0		1.14	0.29	4.45
≥1 times/day	18	36.0	21	42.0		0.86	0.21	3.45
Vegetables, n %					0.07			
1-3 times/month	4	8.0	4	8.0		1.00	-	
1 - 4 times/week	24	48.0	13	26.0		1.89	0.40	8.90
≥1 times/day	22	44.0	33	66.0		0.68	0.15	3.01
Fruits, n %					0.80			
Never	4	8.0	3	6.0		1.00	-	
1 - 3 times/month	6	12.0	5	10.0		0.89	0.13	6.12
1 - 4 times/week	25	50.0	30	60.0		0.62	0.13	3.05
≥1 times/day	15	30.0	12	24.0		0.93	0.17	5.01
Saturated fats, n %					0.08			
Never	42	84.0	33	66.0		1.00	-	
1 - 3 times/month	3	6.0	10	20.0		0.23	0.06	0.92
1 - 4 times/week	5	10.0	7	14.0		0.56	0.16	1.92
Oils (olive, canola), n %					0.003			
Never	17	34.0	8	16.0		1.00	-	
1 - 3 times/month	7	14.0	6	12.0		0.55	0.14	2.18
1 - 4 times/week	18	36.0	11	22.0		0.77	0.25	2.38
≥1 times/day	8	16.0	25	50.0		0.15	0.05	0.48
Fried food, n %					0.26			
Never	7	14.0	7	14.0		1.00	-	
1 - 3 times/month	7	14.0	13	26.0		0.54	0.13	2.17
1 - 4 times/week	31	62.0	22	44.0		1.41	0.43	4.61
≥1 times/day	5	10.0	8	16.0		0.63	0.14	2.91
Carbohydrates/Pastry, n %					0.69			
0 - 3 times/month	1	2.0	2	4.0		1.00	-	
1 - 4 times/week	6	12.0	8	16.0		1.51	0.11	0.85
≥1 times/day	43	86.0	40	80.0		2.15	0.19	24.66
Milk / Yogurt, n %					0.07			
Never	10	20.0	10	20.0		1.00	-	
1 - 3 times/month	7	14.0	13	26.0		0.54	0.15	1.93
1 - 4 times/week	26	52.0	14	28.0		1.86	1.62	5.53
≥1 times/day	7	14.0	13	26.0		0.54	0.15	1.92
Carbonated drinks, n %					0.24			
Never	25	50.0	24	48.0		1.00	-	
1 - 3 times/month	10	20.0	16	32.0		0.60	0.23	1.58
1 - 4 times/week	14	28.0	10	20.0		1.34	0.50	3.60
Coffee/Tea, n %					0.49			
Never	21	42.0	15	30.0		1.00	-	
1 - 3 times/month	5	10.0	7	14.0		0.51	0.13	1.92
1 - 4 times/week	13	26.0	11	22.0		0.84	0.30	2.39
≥1 times/day	11	22.0	16	32.0		0.49	0.17	1.36

*OR and 95% CI were estimated from logistic regression models adjusted for age; OR = 1.00 refers to the reference category.

^†^OR and 95% CI for these variables are expressed per one unit increase.

^‡^The cutoff point for years of quitting smoking was based on the median in controls (27 years).

There was no association between smoking and PrCa (Table 2). The majority of participants (58% of cases and 66% of controls) were never cigarette smokers and 50% of former smokers had quit cigarettes more than 27 years ago. In relation to alcohol use, former drinkers had an OR = 5.2 (95% CI 1.86 - 15.56) for PrCa in comparison to never drinkers. Moreover, cases stopped drinking alcohol on average 10 years more recently then controls (p = 0.008). Nonetheless, there was no clear pattern between alcohol use and PrCa since the proportion of current drinkers and the average number of drinks/week was higher in controls in comparison to cases. We also queried cases and controls about their dietary habits in the year before interview date, and assessed different food items that are consumed in Nigeria (Table 2). The intake of several foods such as vegetables, saturated fats, unsaturated fats (olive and canola oil) and milk or yogurt differed between cases and controls. However, the only statistically significance difference was for unsaturated fats, where men in the highest vs. lowest category had an OR of 0.15 (95% CI 0.05-0.48) for PrCa. There were no clear trends across increasing categories for other food items, which could also be due to small sample size.

The average (SD) age at PrCa diagnosis for the cases was 69 (7.4) years, and the average (SD) elapsed time between PrCa diagnosis and interview date was 0.54 (0.67) months. The majority (76%) of the cases were diagnosed because of symptoms (e.g., urinary frequency, pain, blood in urine) that led them to hospitalization, while 12% of them were diagnosed because of an abnormal digital rectal exam (DRE) or high serum PSA levels (Table 3). We queried both cases and controls regarding PSA and DRE screening for PrCa; 98% of PrCa patients reported having had a serum PSA or digital rectal exam (DRE) before their cancer diagnosis, and the median diagnostic PSA was 72.9 ng/ml and 70% of patients has a PSA > 20 ng/ml. In addition, 32% and 38% of prostate tumors were of Gleason scores of 7 or 8–10, respectively (Table 3). Information on clinical stage of PrCa was not available, because cancer staging is currently not performed in routine clinical practice in Nigeria. However, approximately 90% of the PrCa patients in this study received radiation, hormone therapy or orchiectomy, which are indications for treatment of regional or metastatic tumor stage. In relation to PrCa screening, 80% of controls had never had a serum PSA or DRE (Table 3). In our study, we also measured serum PSA levels in controls from the blood that they provided at the time of interview. Their median serum PSA was 2.13 ng/ml. However, 18% of controls had serum PSA values between 4.0 and 9.9 ng/ml, and 8% had PSA values between 10 and 50 ng/ml, and they were referred for facilitated follow-up in urology.

**Table 3:** **Clinical characteristics, medical comorbidities and information about prostate cancer screening and serum PSA levels in cases and controls**

**Characteristics**	**Cases (n = 50)**		**Controls (n = 50)**	
	**n**	**%**	**n**	**%**
Age at prostate cancer diagnosis (years)				
<50	1	2.0		
50 - 59	4	8.0		
60 - 69	20	40.0		
70 - 79	25	50.0		
Reason for prostate cancer diagnosis				
Abnormal PSA only	1	2.0		
Abnormal PSA or DRE & Symptoms	5	10.0		
Symptoms only	38	76.0		
Don’t know	6	12.0		
Gleason score				
4 - 6	12	24.0		
7	16	32.0		
8 - 10	18	36.0		
Missing	4	8.0		
Serum PSA (ng/dl)*				
<4	1	2.0	36	72.0
4 - 9.9	5	10.0	9	18.0
10 - 19.9	4	8.0	-	-
20 - 49.9	4	8.0	4	8.0
50 - 135	31	62.0	-	-
Missing*	5	10.0	1	2.0
Prostate cancer screening				
No screening (either PSA or DRE)	-	-	40	80.0
PSA only or DRE only	8	16.0	4	8.0
PSA and DRE (both)	41	82.0	6	12.0
Unknown / missing	1	2.0	-	-
Current health				
Excellent	1	2.0	3	6.0
Very Good	1	2.0	9	18.0
Good	20	40.0	21	42.0
Fair	25	50.0	15	30.0
Poor	3	6.0	2	4.0
Comorbid conditions;				
Asthma	1	2.0	3	6.0
High blood pressure	21	42.0	30	60.0
Stroke	1	2.0	2	4.0
Diabetes	6	12.0	7	14.0
High cholesterol	-	-	3	6.0
Ulcers	2	4.0	6	12.0
Chronic back pain	3	6.0	5	10.0
Benign prostatic hyperplasia (BPH)	31	62.0	4	8.0
Urinary tract infections/STD	2	4.0	2	4.0
Use of Traditional Medicine (Current)	11	22.0	12	24.0
Nr of physical exams (past 3 yrs); mean; sd	7.0	11.4	6.2	8.5
Nr of traditional healer visits; mean sd	9.4	17.8	1.7	2.7

*For cases serum PSA indicates the diagnostic PSA, which was abstracted from medical records. For controls serum PSA levels were measured from the blood samples that they provided during in-person interview. One control did not provide a blood sample.

Finally, we compared other comorbid conditions between cases and controls (Table 3). As expected cases reported a higher prevalence of benign prostatic hyperplasia: 62% vs. 8% (p < 0.0001) in comparison to controls. Controls had a higher prevalence of high blood pressure in comparison to cases (60% vs. 42%; p = 0.07), whereas the prevalence of other comorbidities was not significantly different between the two groups. None of the cases or controls reported a history of infectious diseases including HIV/AIDS, hepatitis, syphilis or herpes infection.

## Discussion

Cancer is a major cause of morbidity and mortality worldwide, and its burden is projected to increase by 70% over the next 20 years [34]. The recent World Report on Cancer indicates that by 2030 the majority of the population affected by cancer will be in low- and middle-income countries (LMIC), as they transition towards higher levels of the Human Development Index, with concomitant increasing life span, aging populations, and rapid changes of socio-economic and lifestyle factors [34]. Data from the GLOBOCAN program of IARC show that in Nigeria, PrCa is the leading cancer affecting men, which accounts for 18.2% and 17.7% of all cancer-related diagnoses and deaths, respectively [34,35]. Like other LMIC, efforts in Nigeria to develop cancer prevention and control programs have been hampered by lack of investment in health care infrastructure with multiple competing health priorities. Given that Nigeria is the most populous country in Africa, there is a need to address important gaps in our current knowledge about the etiology of PrCa in Nigerian men, particularly in relation to aggressive forms of this cancer.

The goals of this collaborative study between the Albert Einstein College of Medicine and the University College Hospital in Ibadan, Nigeria, were to assess the feasibility of conducting an epidemiological case–control study of PrCa at the latter institution, and to obtain preliminary insight into PrCa risk factors in Nigerian men. This pilot study demonstrated the ability to ascertain and recruit successfully 50 cases and 50 controls at UCH with very good participation rates of 98% and 93%, respectively. In addition, all participants completed an in-person questionnaire, and 100% of cases and 99% of controls provided blood sample. Our results highlighted the advanced clinical features of PrCa in Nigerian men, and that family history of PrCa and some anthropometric factors (e.g. height, weight and waist circumference) were associated with PrCa risk in this population.

Our pilot data demonstrated that the majority of PrCa patients at UCH were diagnosed at an older age and had more aggressive clinical features (the median diagnostic PSA was 72.9 ng/ml, and 38% had a Gleason score of 8–10 tumor), in comparison to PrCa patients diagnosed in the US or other developed countries [2]. Information on clinical stage of PrCa was not available. However, approximately 90% of our PrCa patients might have had regional or metastatic tumor at diagnosis because they were treated with radiation and/or hormone therapy. A limitation is that tumor staging is not performed routinely in clinical practice in Nigeria. Thus, implementation of clinical staging by urologists and pathologists would better characterize not only the clinical features of PrCa but also help improve decision-making regarding PrCa treatment. Our results are consistent with those of several other case reports [37,39-43] showing that 65%-80% of all PrCa patients in Nigeria are diagnosed with advanced tumor grade and stage. In addition, a recent community-based PrCa awareness program, which was conducted among 4,110 men aged 40 years or older in Lagos, a major urban center near Ibadan, reported that 56%, 28% and 35% of 43 men diagnosed with PrCa through this program had a diagnostic PSA ≥20 ng/ml, Gleason score 8–10, and metastatic disease, respectively [41].

Collectively these data indicate that either PrCa is more aggressive among African men or there is a lack of PrCa screening programs, which would shift the clinical features of this cancer. Although 98% of our PrCa patients reported having had a serum PSA or digital rectal exam (DRE) before their cancer diagnosis, these are likely diagnostic tests rather than preventive screening tests. Data from our controls showed that 80% of men had not had any PSA or DRE tests done, indicating the lack of PrCa preventive screening programs in Nigeria. The issue of PrCa screening is the subject of ongoing debate, especially after the U.S. Preventive Service Taskforce recommended against PSA screening in 2012 (D grade) [45]. While this decision considered many aspects of PSA screening including over-diagnosis and overtreatment of PrCa, the recommendation was based on conflicting results from two large PrCa screening trials conducted in the US [46,47] and Europe [48,49]. The PLCO trial in the US did not report any benefit of PSA screening on PrCa-specific mortality [46,47], while the European trial reported a statistically significant 21% reduction in PrCa-specific mortality associated with PSA screening at 13 years of follow-up [48,49]. However, a limitation of both trials is that they did not address the benefit of screening in high-risk populations, namely men of African descent or those with a family history of PrCa. While there is an ongoing discussion to implement shared decision making between primary care physicians and their patients about risks and benefits of screening in developing countries, such as the U.S. [50], the current screening guidelines for Nigeria and other LMIC in Africa are less clear. This suggests that further studies are needed to assess the benefits of PSA screening in African men, and those with family history of PrCa. Although our study was not designed to address this issue, we note that some discussion about PrCa screening in Nigeria needs to be implemented between patients and health care practitioners or physicians.

In our pilot study we also collected information on various risk factors for PrCa in Nigerian men. Consistent with other PrCa studies [10,11], a first-degree family history of PrCa was associated with a 4.9-fold increased risk (95% CI 1.0 - 24.8) of PrCa in our data. However, self-reported family history for second-degree relatives was limited. The accuracy of self-reported cancer in relatives depends largely on family size as well as knowledge about cancer diagnosis in relatives. Although the average family size is relatively large in Nigeria, knowledge about PrCa or family history of this disease might be limited for several reasons including undiagnosed cancer, low health literacy about PrCa, as well as social stigma around cancer in Nigerians. For example, in a cross-sectional study of 156 urban men in Nigeria, Ajape et al. [51] reported that 79% and 94% of them, respectively, had never heard any information on PrCa or PSA screening. Thus it is important to raise awareness on PrCa among Nigerian men.

With regard to anthropometric factors, although we observed statistically significant inverse associations between risk of PrCa and height, weight and waist circumference, there was no association with body mass index. The inverse association with weight may be due to the fact that the proportion of men who had lost 5 kg or more in the past year was much higher in cases in comparison to controls (52% vs. 16%; p < 0.0001), which could be attributed to cachexia due to cancer. For lifestyle and dietary factors we did not find any clear trends or associations with PrCa risk. However, the sample size of this study was very small and we had limited power for comprehensive data analyses. Larger epidemiological studies will be needed for more definitive investigation of the association between several lifestyle and dietary factors and risk of PrCa in this population.

Some feasibility issues to be considered for large epidemiologic studies in hospital settings in Africa include the number of PrCa patients that are seen on an annual basis, potential delays in recruitment of patients, and the infrastructure for processing and long term storage of blood/ serum samples, communication issues, and challenges with abstracting data from medical charts. The UCH sees on average 140 PrCa patients per year. Thus for large studies (e.g. 500 cases/500 controls) a 4 to 5- year recruitment period is needed in order to achieve the required sample size, assuming ≥80% participation rate. Our study was delayed due to strikes that affected hospital administration, which could potentially affect larger studies. There were limitations surrounding infrastructure in laboratories and bio-specimen storage. In our study we extracted DNA soon after blood collection and DNA samples are currently stored at −20°C in different laboratories at UCH. However, in order to conduct large-scale epidemiological studies among thousands of patients, there is a need to build a central biorepository, which could be used for long-term storage of DNA, serum or plasma samples at −80°C for future research, as is done in most developed countries. We also encountered difficulties in communication via phone or internet between the research teams in Ibadan and New York. Regular phone or Skype communication on a bi-weekly or monthly basis was necessary to coordinate different research activities and maintain collaboration, and the quality of these interactions was impaired when the connection was poor. Finally, secondary extraction of clinical and laboratory data from medical charts for some patients was challenging due to missing detailed reports on medical charts. Thus, establishment of an electronic medical records system will be very useful in capturing the minimum clinical/pathological data for PrCa that can be used for research.

As with other case–control studies, another limitation of our study includes the potential for selection bias and recall bias. The UCH is a major tertiary hospital in Ibadan and thus patients who are admitted to this hospital might not be representative of the entire general population i.e., the referral filter bias. Since there is no national health insurance program in Nigeria and most hospital expenses are paid out of pocket, there is a possibility that both cases and controls who are hospitalized at UCH are different from men in the general population particularly those in the rural areas, and thus, the generalizability of the results may be limited. Another potential bias could be misclassification of outcome since some of the controls with high PSA levels may have had underlying PrCa. Five out of nine controls with serum PSA values between 4 and 10 ng/ml reported having had either a benign enlargement of their prostate (BPH) or urinary tract infection. Although all controls with serum PSA >4 ng/ml were referred for additional follow-up with Urology, we were not able to ascertain what proportion of these controls had underlying PrCa. In a large community-based PrCa awareness study in Lagos, the prevalence of PrCa among 4,110 men aged >40 years was 1.1% [41]. However, another large population-based PrCa screening study of 1,037 healthy men aged 50 to 74 years in Ghana [52] reported a higher PrCa prevalence of 7%. Thus, we anticipate that one to three controls in our study may potentially have had an underlying PrCa, a consequence of which would have been to bias case–control differences towards the null. In order to address potential outcome misclassification we conducted a sensitivity analysis by excluding controls with a serum PSA >10 ng/ml (n = 4), who might possibly have had an underlying PrCa. In these analyses, associations between PrCa risk and either family history of PrCa or anthropometric, demographic and lifestyle characteristics were similar to those observed using all controls (data not shown). Lastly, as in studies elsewhere, participants in our study might have misreported sensitive information, such as infectious diseases, as none of the participants reported HIV/AIDS, hepatitis, syphilis or herpes infection.

## Conclusions

This pilot study of PrCa demonstrated the ability to ascertain and recruit participants as well as to collect data successfully at UCH in Ibadan, Nigeria. Our results highlighted the advanced clinical characteristics of PrCa in Nigerian men, and that family history of PrCa and some anthropometric factors were associated with PrCa risk in this population. This study also represents a small step forward in addressing the need to build collaborative research partnerships in cancer. The ultimate objective of our study is to establish and sustain a platform for continual development of research and clinical capacities for PrCa research. As part of this collaboration we (UCH and Einstein) are now part of the Men of African Descent and Carcinoma of the Prostate Consortium [9,53], which aims to undertake research that will improve understanding of the etiology, genetics, prevention, and treatment of PrCa in Africa.

## Abbreviations

CI: Confidence interval
DRE: Digital rectal examination
IARC: International Agency for Research on Cancer
OR: Odds ratio
MADCaP: Men of African Descent and Carcinoma of the Prostate Consortium
PrCa: Prostate cancer
PSA: Prostate specific antigen
UCH: University College Hospital
